# Low-grade fibromyxoid sarcoma of the vagina: A tumor, not previously reported at this site

**DOI:** 10.4274/tjod.73626

**Published:** 2014-09-15

**Authors:** Nilgün Güdücü, İpek Çoban, Nuray Başsüllü, Gökçenur Gönenç, Kılıç Aydınlı

**Affiliations:** 1 İstanbul Bilim University Faculty of Medicine, Department of Obstetrics and Gynecology, İstanbul, Turkey; 2 İstanbul University Cerrahpaşa Faculty of Medicine, Department of Obstetrics and Gynecology, İstanbul, Turkey; 3 İstanbul Bilim University Faculty of Medicine, Department of Pathology, İstanbul, Turkey

**Keywords:** Low-grade fibromyxoid sarcoma, vagina, mesenchymal

## Abstract

This report presents the first case of low-grade fibromyxoid sarcoma (LGFMS) arising from the vaginal wall (a rare soft-tissue sarcoma of subfascial planes) and draws attention to differential diagnosis of masses arising from the vaginal wall. A patient presenting with abdominal pain to emergency department was diagnosed to have an ovarian mass filling the Douglas space. At laparoscopy, the origin of the mass was identified as the posterior vaginal wall. After vaginal excision of the gelatinous mass, pathologic diagnosis revealed a rare tumor, LGFMS. We discussed the differential diagnosis of vaginal LGFMS.

## INTRODUCTION

Low-grade fibromyxoid sarcoma (LGFMS) is a rare soft-tissue sarcoma that presents in subfascial planes as an isolated tumoral mass and it was first described by Evans in 1987^(1)^. The specious benign appearance may end up with local recurrence or metastasis at an unpredictable time in the future. LGFMSs are mostly described as tumors of trunk and lower extremities. To the best of our knowledge there is no LGFMS of the vagina reported before, literature review revealed two vulvar and one ovarian LGFMS^(2,3)^. This report aimed to present the first LGFMS arising from the vaginal wall and to draw attention to differential diagnosis of future masses arising from the vaginal wall.

## CASE

**Figure 1:**
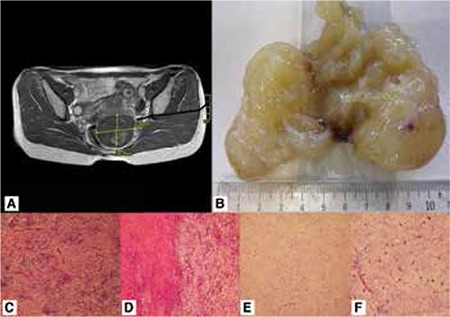
Figure 1A. MRI of the mass filling Douglas space and protruding into posterior vaginal wall Figure 1B. Macroscopically yellow-white colored, gelatinous mass Figure 1C. Myxoid areas revealed a rich capillary network Figure 1D. Alternating areas with fibrous (left) and myxoid (right) stroma are apperant Figure 1E. Myxoid zone of the lesion is of low cellularity and omposed of bland spindle shaped cells Figure 1F. At high power, the tumor cells were deeptively bland and some of the cells exhibited a stellate morphology. Mitotic figures were scarce

A 36 years old woman presented with abdominal pain to emergency department of a private hospital. Her magnetic resonance imaging report revealed a 6 cm ovarian cyst (Figure 1A). She was referred to an outpatient gynecology clinic after exclusion of acute abdomen. She went to her own gynecologist, where she had her last gynecologic examination and transvaginal ultrasound 14 months ago with completely normal findings. At the outpatient clinic a mass filling Douglas space and protruding into the posterior vagina was easily recognized. Transvaginal ultrasonography revealed a septated ovarian mass. She was scheduled to laparoscopic ovarian cystectomy in our hospital. She was prepared in gynecologic position for laparoscopy, after the introduction of the umbilical trocar, inspection revealed normal appearing ovaries and a mass filling Douglas space and most probably arising from the posterior vaginal wall. We decided to end laparoscopy and proceed with vaginal excision of the mass. We made an incision on the mass protruding to posterior vaginal fornix and easily extracted a semisolid, partly encapsulated gelatinous mass. The encapsulated mass measured 9x6x4 cm. Macroscopically the tumor had a yellow-white gelatinous form and glistening appearance giving the impression of a myxoid substance accumulation (Figure 1B). Microscopicallly the tumor had fibrous and myxoid areas, a swirling growth pattern with benign appearing fibroblastic spindle cells dispersed inbetween, there were a few mitotic figures, low-to-moderate cellularity and mild nuclear atypia, there were no necrotic areas and nuclear pleomorphism was slight, the capillary network was rich, the capsule was fragmented and it was impossible to determine positivity of the surgical margins (Figure 1C, 1D, 1E, 1F). Immunohistochemical staining with S-100 was performed. The diagnosis was LGFMS of the vaginal wall and the patient was referred to radiotherapy.

## DISCUSSION

Differential diagnosis of vaginal mass lesions includes cysts arising from müllerian remnants. Most of these cystic lesions are benign and they grow anteriorly towards bladder, but aberrant embryological development may leave remnants of Gartner’s duct anywhere in the vagina and Gartner’s duct cysts in posterior vaginal wall has been reported previously^(3)^. Most of these cystic lesions are asymptomatic and are diagnosed during routine gynecologic examination and do not require any intervention. Malignant transformation of Gartner’s duct cysts are rare^(4^). Other cystic malignant lesions arising from the vaginal wall are also rare and include vaginal squamous cell carcinoma^(5)^. Mesenchymal lesions of the vagina pose a diagnostic challenge to the pathologist and include leiomyoma, leiomyosarcoma, schwannoma, angiomyofibroblastoma, aggressive angiomyxoma, cellular angiofibroma, superficial myofibroblastoma of the lower female genital tract and gastrointestinal stromal tumors^(6,7)^. A mass lesion in Douglas pouch with a similar clinical presentation to our case was reported as adenoma malignum^(8)^. Differential diagnosis of mass lesions presenting in the lower parts of the vagina may also include cysts of Bartholin’s gland. In the differential diagnosis of most of the lesions immunohistochemistry was reported to have a limited role and the diagnosis mainly depended on morphology^(9)^.

The gynecological examination of our case was completely normal 14 months ago, but preoperative duration of LGFMS has been reported to range from 1 month to 25 years^(10)^. The case presented here is of additional interest and importance because of its location. Previously LGFMS cases have been reported from vulva and ovary but this will be the first case reported to develop from the vaginal wall.

The largest case series of LGFMS reported by Evans were treated with excision initially, local recurrences and metastases were also mostly treated by excision^(10)^. They sometimes used adjunctive radiotherapy and chemotherapy. Our case has been recently diagnosed and operated, therefore we cannot give data regarding disease free survival, but previously reported deaths due to tumor recurrence occurred at a range of 3 to 42 years, also the author reported survival with tumor upto 70 years^(10)^.

In conclusion, most of the mass lesions of vagina are benign lesions but malignancies including LGFMS must be remembered in the differential diagnosis. These tumors have a highly variable overall prognosis and avoidance of intraabdominal operation may affect survival.
